# Long-lasting insecticide-treated house screens and targeted treatment of productive breeding-sites for dengue vector control in Acapulco, Mexico

**DOI:** 10.1093/trstmh/tru189

**Published:** 2015-01-19

**Authors:** Azael Che-Mendoza, Guillermo Guillermo-May, Josué Herrera-Bojórquez, Mario Barrera-Pérez, Felipe Dzul-Manzanilla, Cipriano Gutierrez-Castro, Juan I. Arredondo-Jiménez, Gustavo Sánchez-Tejeda, Gonzalo Vazquez-Prokopec, Hilary Ranson, Audrey Lenhart, Johannes Sommerfeld, Philip J. McCall, Axel Kroeger, Pablo Manrique-Saide

**Affiliations:** a Servicios de Salud de Yucatán, Gobierno del Estado de Yucatán, Mérida C.P. 97000, Mexico; b Universidad Autónoma de Yucatán, Mérida C.P. 97000, Mexico; c Servicios Estatales de Salud de Guerrero, Chilpancingo C.P. 39090, Mexico; d Centro Nacional de Programas Preventivos y Control de Enfermedades, Secretaria de Salud, Mexico; e Vector Biology Department, Liverpool School of Tropical Medicine, Liverpool L3 5QA, UK; f Department of Environmental Sciences, Emory University, Atlanta, GA, 30322, USA; g US Centers for Disease Control and Prevention, Atlanta, GA30329, USA; h Special Programme for Research and Training in Tropical Diseases (TDR), World Health Organization, 1211 Geneva 27, Switzerland

**Keywords:** *Aedes aegypti*, Control, Dengue, LLIS, Mexico, Targeted treatment

## Abstract

**Background:**

Long-lasting insecticidal net screens (LLIS) fitted to domestic windows and doors in combination with targeted treatment (TT) of the most productive *Aedes aegypti* breeding sites were evaluated for their impact on dengue vector indices in a cluster-randomised trial in Mexico between 2011 and 2013.

**Methods:**

Sequentially over 2 years, LLIS and TT were deployed in 10 treatment clusters (100 houses/cluster) and followed up over 24 months. Cross-sectional surveys quantified infestations of adult mosquitoes, immature stages at baseline (pre-intervention) and in four post-intervention samples at 6-monthly intervals. Identical surveys were carried out in 10 control clusters that received no treatment.

**Results:**

LLIS clusters had significantly lower infestations compared to control clusters at 5 and 12 months after installation, as measured by adult (male and female) and pupal-based vector indices. After addition of TT to the intervention houses in intervention clusters, indices remained significantly lower in the treated clusters until 18 (immature and adult stage indices) and 24 months (adult indices only) post-intervention.

**Conclusions:**

These safe, simple affordable vector control tools were well-accepted by study participants and are potentially suitable in many regions at risk from dengue worldwide.

## Introduction

The dengue vector *Aedes aegypti* is a highly anthropophilic, endophilic and endophagic mosquito and has successfully exploited human-made ecosystems more than any other vector. Traditional *Ae. aegypti* interventions that are based on insecticide application such as indoor or outdoor space-spraying (or fogging) and larviciding, although effective in some settings, have shown limitations in terms of spatial and temporal coverage, residual power, sustainability and effectiveness in many contexts.^1^ There is a pressing need from vector control programmes worldwide, for better dengue vector control tools that can achieve sustained reduction of dengue virus transmission by impacting the adult vector populations and/or interrupting their interaction with humans.^2^

Ecosystem management interventions such as the deployment of insecticide treated materials (ITMs) as window/indoor net curtains in houses, and the targeted treatment (TT) of productive breeding-sites have shown potential for integrated dengue vector control in many geographical contexts.^1,3–6^ Long-lasting insecticidal net screens (LLIS) are factory-produced mosquito nets pre-loaded with synthetic pyrethroid insecticide that is intended to retain its biological activity for at least 20 standard washes under laboratory conditions and 3 years of recommended use under field conditions.^7^ Deployed as bednets, LLIS potentially can impact vector longevity at both household and community levels by reducing human biting rates.^4^ Encouraging results have also been shown when LLIS are deployed as window or door curtains or as water jar covers for dengue control, particularly in Latin America^5,6^ though the magnitude of such effect was sometimes dependent on the coverage attained, which could decline rapidly over time.^5^ Targeting treatment of productive breeding-sites is a strategy that aims to impact vector populations by treating only water containers that produce the greatest number of pupae,^8^ and also has potential for effective community-level dengue control.^1,9,10^

There is a need for more studies to comprehensively assess the long-term impact and cost-effectiveness of LLIS and TT in controlling local mosquito populations and reducing dengue transmission, particularly if both could be deployed simultaneously. The present study aimed to assess the long-term (over 2 years) impact of LLIS and TT in controlling domestic *Ae. aegypti* infestations, when deployed simultaneously, in an urban environment with perennially high dengue transmission in Mexico. The data reported here build upon the findings of the initial phase of a cluster randomized trial which investigated the impact of LLIS alone on adult vector indices.^11^

## Materials and methods

This study formed part of a multi-country effort with a universal initial core protocol developed during a TDR-IDRC proposal development workshop in 2009. An earlier situational analysis of randomly selected study clusters (neighborhoods) in urban environments,^12^ provided the initial information on which this intervention study was designed.

### Study site

**Figure 1.**
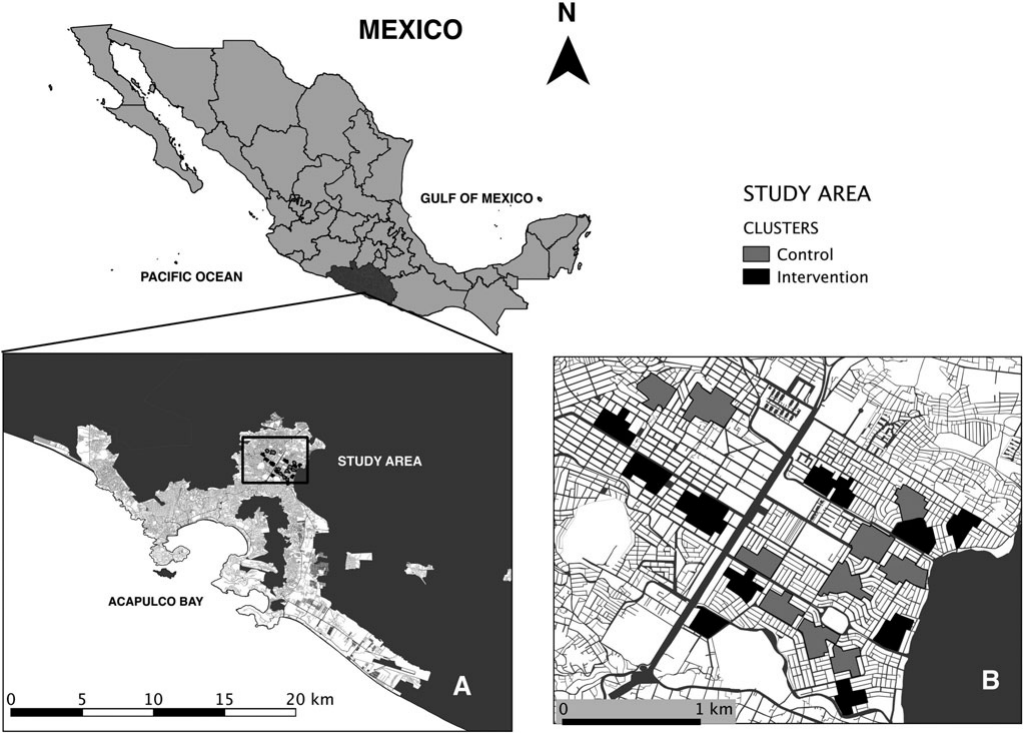
Study site. Location of Acapulco in Mexico and (A,B) the particular study area within Acapulco city showing the distribution of the study clusters with and without interventions.

Ciudad Renacimiento (here after called Renacimiento) in Acapulco, is in Guerrero state, Mexico (Figure 1). Guerrero has one of the highest levels of dengue in Mexico, Acapulco reported >30% of the total dengue cases in Guerrero in the last decade.^13^ Renacimiento is a high-risk area for dengue transmission: in 2011, entomological surveys found adult Aedes in 40% and 85% of houses during the dry and rainy seasons respectively, and over 70% of houses had incomplete walls, unprotected windows and/or open doors during daytime; although all households reported receiving water supply, 98% stored water in tanks (1000 litres) or barrels (100–200 litres), which produce 89% of total pupae in the study area.^12^ Such large and highly productive containers are the focus of a targeted control effort by the local ministry of health (MoH) using larvicide (Temephos).

### Study design

A cluster-randomized sampling design with cross-sectional entomological surveys^12^ was performed in 20 geographic clusters of 100 households each, with 10 randomly assigned to either intervention or control treatments, over 24 months. Procedures for selection of clusters, random assignment of treatments and statistical power calculations were described previously.^11^

### House screening with LLIS

**Figure 2.**
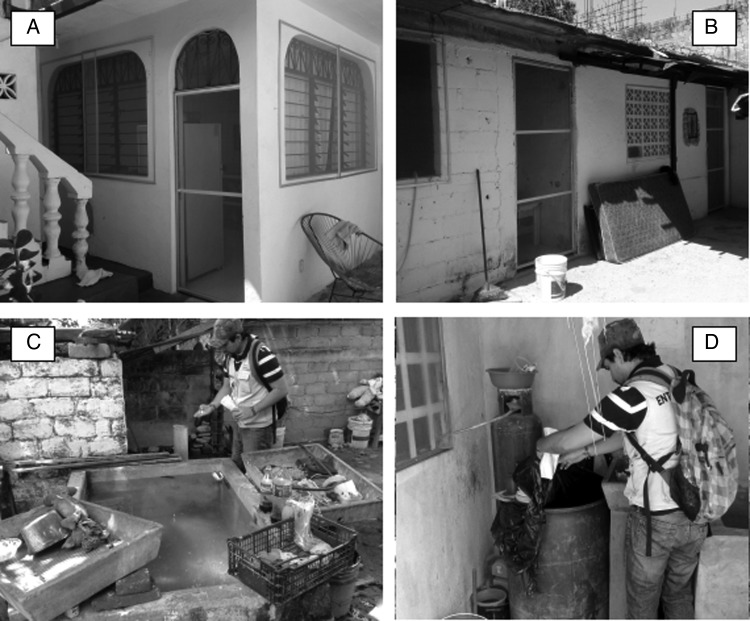
Photographs show (A,B) the long-lasting insecticidal net screens ([LLIS]; Duranet^®^ [Clarke Mosquito Control, Roselle, IL, USA]) mounted on aluminum frames and fixed to windows and external doors of treated houses and (C,D) the targeted treatment (TT) of the most productive *Aedes aegypti* breeding sites with the larvicide Natular^®^ DT (Spinosad 7.48%) in Acapulco Mexico.

Duranet^®^ screens (0.55% w.w. alpha-cypermethrin-treated non-flammable polyethylene netting [145 denier; mesh=132 holes/sq. inch]; Clarke Mosquito Control, Roselle, IL, USA; WHOPES approved for LLIS use) were mounted in aluminum frames custom-fitted to doors and windows of residential houses (Figure 2 A,B). The installation in 586 households from nine intervention clusters, carried out in collaboration with a local small business and the MoH, started in April 2012 and was finished by August 2012. In January 2013 the coverage of intervention was 78.0% of the households (780/1000) in the intervention clusters. During the installation, at least one person in every household received information on the use and maintenance of the LLIS through person-to-person communication.

### Targeted treatment of the most productive *Ae. aegypti* breeding sites

Targeted treatment to prevent *Ae. aegypti* breeding in the most productive sites, was implemented 14 months after the beginning of LLIS installation (June 2013). All 1789 water tanks and 200 litre drums/barrels (Figure 2 C,D) in the households of intervention clusters, which were the most productive type of containers in baseline pupal surveys, were treated with the environmentally friendly larvicide Natular^®^ DT (Spinosad 7.48%; Clarke Mosquito Control; WHOPES approved), delivering 1 tablet per 200 litres. The first cycle of application was performed at the end of the dry season in 2013 (September, n=1791 tanks and barrels) and was repeated every two months until March 2014 (November 2013 n=1686, January 2014 n=1658, March 2014 n=1595).

No interventions were delivered to the control clusters. However, existing routine vector control activities the local vector control program continued in both intervention and control clusters throughout the study. These included adulticiding (outdoor and indoor spraying with Chloropyrifos and Propoxur, respectively) and larviciding (Abate and Spinosad) in response to elevated dengue and entomologisal risk indices.^14^ Notable emergency vector control activities occurred in February to April 2013 with a breeding-site reduction campaign all over the city called ‘Megaoperativo’ in July and ULV spraying from vehicles and airplanes in September 2013 (after tropical storms ‘Manuel’ and ‘Ingrid’).

### Entomological surveillance

Seven cross-sectional entomological surveys were conducted in treatment and control clusters: before (March 2011, September 2011, March 2012) and at 5, 12, 18 and 24 months (September 2012, March 2013, October 2013, March 2014; wet, dry, wet and dry seasons, respectively) post-intervention.

#### Indoor adult mosquito surveys

Adult entomologic surveys were performed in a sub-sample of 32 houses from each cluster. The houses were randomly selected in each cross-sectional survey during each entomologic survey date. Indoor adult mosquitos were collected using modified CDC backpack aspirators (John W. Hock Company, Gainesville, FL, USA) for 15 minutes per house. Collections within each cluster were performed on the same day between 09:00-15:00 hrs. All mosquitoes collected were identified for species and sex and kept in vials for future use.

#### Larval and pupal surveys

Larval and pupal surveys were conducted in all 2000 houses from the 20 study clusters for each entomologic survey according to a standard protocol.^15^ Intradomestic and peridomestic spaces of residential premises were inspected and only water holding containers were examined. Containers were classified according to type, source of water, capacity, presence of a functional lid, proximity to vegetation, and presence of larval control measures. All immatures were collected except in large containers, where a sample of pupae was collected and a correction factor applied.^15^ In the laboratory, a sub-sample of 10% of pupae was allowed to develop into adult mosquitoes to identify their species and sex.

### Data management and statistical analysis of entomological indicators

From indoor adult collections we recorded: houses positive for female *Aedes*; houses positive for blood-fed female *Aedes*; houses positive for male *Aedes*; number of female *Aedes* per positive house; number of blood-fed female *Aedes* per positive house; and number of male *Aedes* per positive house. From immature collections we recorded: houses positive for immature (larva and pupae) *Aedes*; houses positive for *Aedes* larvae; number of *Aedes* larvae per house; houses positive for *Aedes* pupae; number of *Aedes* pupae per house; and pupae per person: number of *Aedes* pupae/number of inhabitants of a household.

The three classic *Stegomyia* indices: the container index (CI), representing the (number of containers with *Ae. aegypti* immatures/wet containers inspected)×100; the house index (HI), representing the (number of houses with *Ae. aegypti* immatures/houses inspected)×100; the Breteau index (BI), representing the number of containers positive for *Ae. aegypti* immatures/houses inspected)×100; and the pupae per person index (PPI) which is the ratio between pupae and persons living in each cluster were computed at the cluster level (Table 1). The difference between control and treatment clusters across the seven survey dates were evaluated with Mann-Whitney non-parametric tests.

**Table 1.** Comparison between treated (intervention [I]) and untreated (control [C]) groups on classic Stegomyia indices and pupal indicators at the cluster level in Acapulco, Guerrero

	Dry 2011		Rainy 2011		Dry 2012		Rainy 2012		Dry 2013		Rainy 2013		Dry 2014	
	I	C	I	C	I	C	I	C	I	C	I	C	I	C
CI	0.72	0.64	4.38	4.5	3.56	3.08	1.47	1.65	1.33	1.79	0.86*	1.82*	1.11	1.51
HI	4.40	4.80	20.45*	26.53*	16.21	15.42	7.58	9.38	6.28	9.00	4.36*	9.70*	4.10	5.90
BI	5.50	5.30	31.75	36.5	20.43	19.16	9.18	11.21	7.31	10.80	4.49*	10.40*	5.00	7.00
PPI	0.03	0.03	0.20	0.21	0.17	0.15	0.04	0.05	0.03*	0.10*	0.023*	0.105*	0.018*	0.071*

BI: Breteau index; CI: container index; HI: house index; PPI: pupae per person index.

^*^ Mean of indicators followed by an asterisk symbol indicates significant difference between treated (I) and untreated (C) groups (p<0.05, Mann-Whitney non-parametric tests).

Logistic regression models (for presence-absence data) and negative binomial models (for count data) accounting for each house membership in a given sampling cluster were performed for each cross-sectional entomological evaluation survey as described in Manrique-Saide et al.^11^. Odds ratios (OR) and incidence rate ratios (IRR) with 95% CIs were assessed and significance expressed at the 5% level. A generalized additive mixed model (GAMM) was applied to determine the association between various household-level entomologic indicators and the time (in days) since the installation of the LLIS. Time to intervention (t_i_) was calculated by estimating the number of days that elapsed between the installation of the LLIS and the entomologic survey of each treatment house. We excluded the control houses from this analysis because analyses aimed at quantifying the temporal effect of LLIS. The full model had the form: $Y_{Aedes}=\alpha +f(t_{i})+Z(cluster_{i})+\epsilon_{i}$. Where *Y_Aedes_* is the entomologic measure and $Z(cluster_{i}),\epsilon_{i}∼N(0,\sigma^{2})$, represents a random effects term associated with observations from the same cluster. We used a negative binomial or binomial link functions depending if *Y_Aedes_* was based on counts or binary values, respectively. We quantified the (possibly) non-linear relationship between the response variable and time since LLIS installation by incorporating a smoothing function ($f\;(t_{i})$) representing the additive component [1]. We fitted $f\;(t_{i})\;\;$by applying a penalized cubic spline function to the data [1]. We assessed the importance of time since the installation of LLIS by evaluating the significance of the $f\;(t_{i})$ term. Akaike Information Criterion (AIC) scores were used to compare the full model with a GAMM model without random effects. A model with ΔAIC=2 or more units lower than any other model was considered the best. Once the best model was identified, we plotted each predicted $f\;(t_{i})$ as either a curve (if $f\;(t_{i})$ was significant) or a line (if $f\;(t_{i})$ was not significant). Analyses were performed using STATA 12.0 (Stata Corp, College Station, TX, USA) and the mgcv package from the R statistical software (Foundation for Statistical Computing, Vienna, Austria).

### Ethical aspects

This study received clearance from the ethical Committee of the Mexican Ministry of Health of Guerrero and the ERC (Ethical Review Committee) of WHO. Written informed consent was obtained for each participating household.

## Results

### Impact of house screening with LLIS

**Figure 3.**
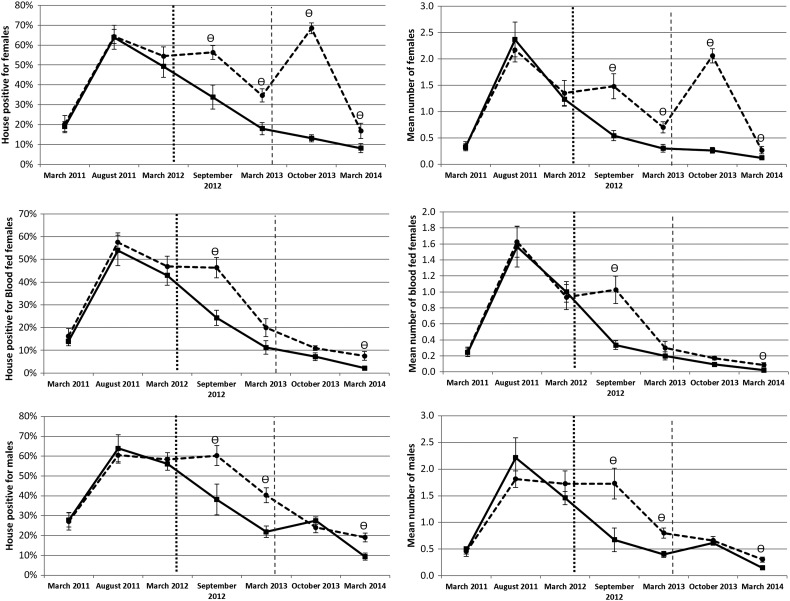
Comparison between treated (solid line) and untreated (broken line) groups of percentage of infested houses (left) and infestation density (right) for *Aedes aegypti* in Acapulco, Guerrero. The vertical dotted and dashed lines represent the start of long-lasting insecticidal net screens (LLIS) and targeted treatment (TT) interventions, respectively. The symbol denotes dates when the index was significantly different between treated and control groups on that date. Error bars show the standard error of the mean.

During the first 12 months of the study, treatment clusters received only LLIS. The preliminary findings from this part of the study have been reported earlier.^11^ At five months post-intervention with LLIS, significantly fewer treated houses were infested with *Ae. aegypti* adult females (OR=0.38, 95% CI: 0.21–0.69), blood-fed females (OR=0.36, 95% CI: 0.21–0.60) and males (OR=0.39, 95% CI: 0.19–0.77). A significant impact was still seen at 12 months post-intervention for adult females (OR=0.41, 95% CI: 0.25–0.68) and males (OR=0.41, 95% CI: 0.27–0.64) but not for blood-fed females (OR=0.51, 95% CI: 0.24–1.05) (Figure 3). Analyses of infestation density showed a similar trend with a significant reduction in mean *Ae. aegypti* abundance in houses with LLIS: adult females at 5 (IRR=0.37, 95% CI: 0.27–0.49) and 12 (IRR=0.40, 95% CI: 0.23–0.70) months post-intervention; males at 5 (IRR=0.39, 95% CI: 0.28–0.54) and 12 (IRR=0.49, 95% CI: 0.33–0.72) months; blood-fed females at 5 (IRR=0.32, 95% CI: 0.23–0.45) but not at 12 (IRR=0.49, 95% CI: 0.23–1.05) months (Figure 3).

**Figure 4.**
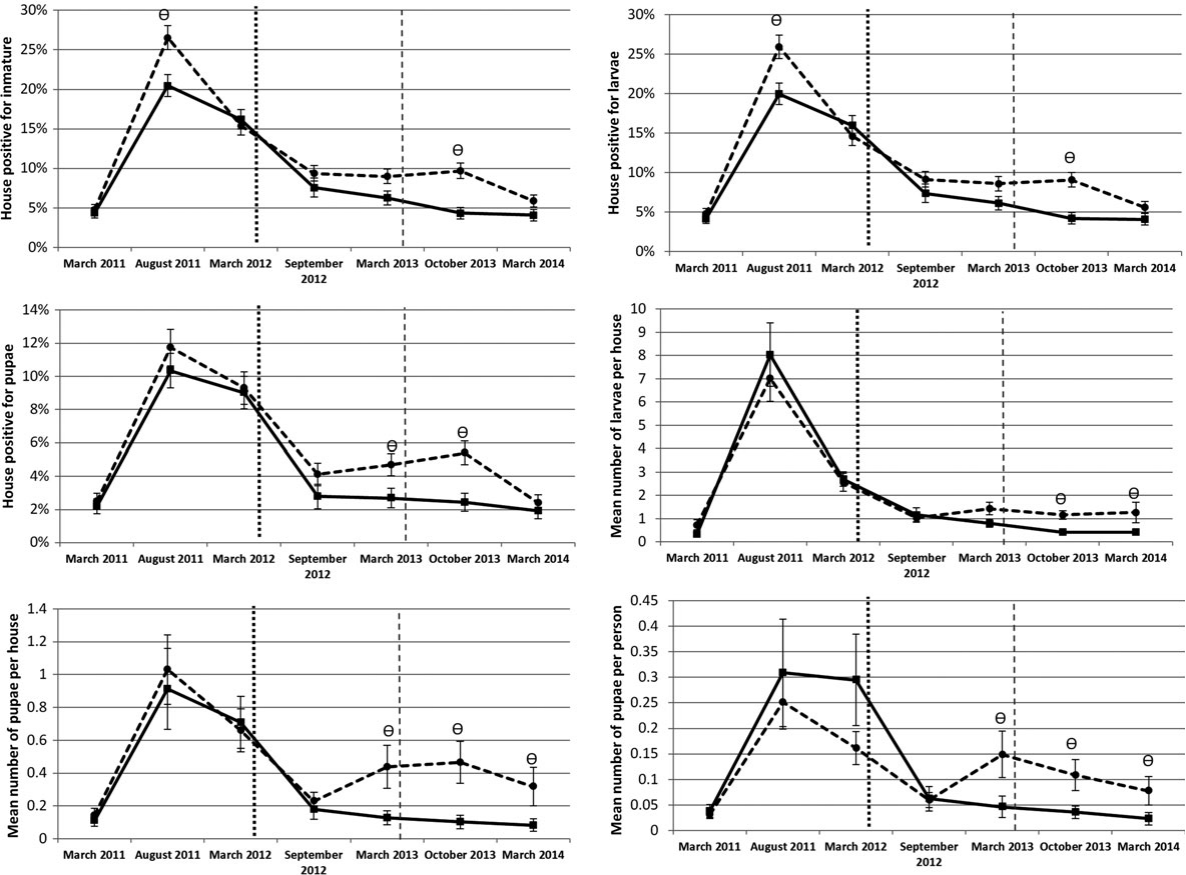
Comparison between treated (solid line) and untreated (broken line) groups of *Aedes aegypti* immature-based indicators for in Acapulco, Guerrero. The vertical dotted and dashed lines represent the start of long-lasting insecticidal net screens (LLIS) and targeted treatment (TT) interventions, respectively. The symbol denotes dates when the index was significantly different between treated and control groups on that date. Error bars show the standard error of the mean.

At 5 months post-intervention with LLIS only, no significant differences between treated and untreated houses were observed in the immature-based indicators (Figure 4). However, a significant impact was seen at 12 months post-intervention with LLIS for all pupae-based indicators (as a proxy for adult vectors): i.e., houses positive to *Aedes* pupae (OR=0.56, 95% CI: 0.33–0.96), number of *Aedes* pupae per house (IRR=0.29, 95% CI: 0.12–0.70) and pupae per person (IRR=0.31, 95% CI: 0.11–0.86). When analysing larval-based indicators these were lower in intervention clusters after the intervention compared to control clusters, but the differences were not statistically significant (Figure 4).

**Figure 5.**
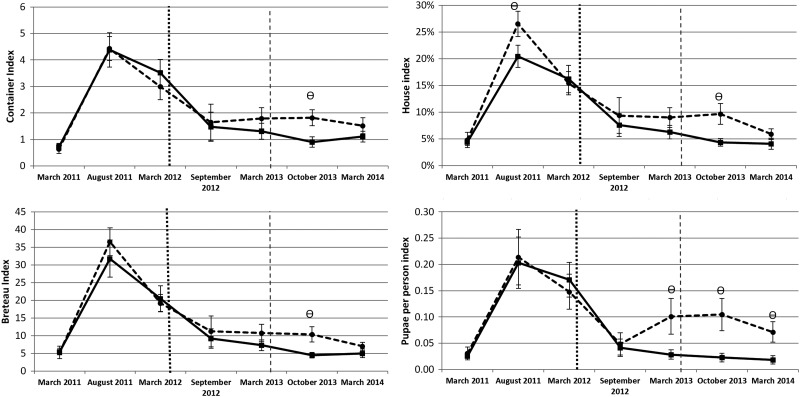
Comparison between treated (intervention) and untreated (control) groups on classic *Stegomyia* indices and pupal indicators at the cluster level in Acapulco, Guerrero The vertical dotted and dashed lines represent the start of long-lasting insecticidal net screens (LLIS) and targeted treatment (TT) interventions, respectively.

At baseline, all the *Stegomyia* indices and PPI were similar between both intervention and control groups (Figure 5). Five months after the installation of LLIS, indices showed a slight decrease in the intervention clusters (Figure 5). At 12 months, only water-holding containers and containers positive for pupae were significantly different between intervention and control clusters (Figure 5).

### Impact of the combination of LLIS and TT

The impact of both approaches was assessed at 18 and 24 months, following introduction of TT at 14 months post-intervention. At 18 months post-intervention, significantly fewer treated houses were infested with *Ae. aegypti* adult females (OR=0.07, 95% CI: 0.05–0.10), but not with blood-fed females (OR=0.63, 95% CI: 0.36–1.09) or males (OR=1.19, 95% CI: 0.84–1.7) (Figure 3). At 24 months post-intervention, significantly fewer adult females (OR=0.44, 95% CI: 0.20–0.95), blood-fed females (OR=0.28, 95% CI: 0.10–0.74) and males (OR=0.44, 95% CI: 0.27–0.71) were found in treated houses.

Analyses of infestation density based on adult catches showed a similar trend with a significant reduction in adult females (IRR=0.12, 95% CI: 0.08–0.19) at 18 months post-intervention; but not for blood-fed females (IRR=0.54, 95% CI: 0.29–1.0 or males (IRR=0.93, 95% CI: 0.72–1.22) (Figure 3). At 24 months post-intervention, significantly lower numbers of indoor adult females (IRR=0.04, 95% CI: 0.21–0.98); blood-fed females (IRR=0.25, 95% CI: 0.09–0.70) and males (IRR=0.48, 95% CI: 0.27–0.86) were found in treated houses.

Houses in treated clusters also had significantly lower immature infestation levels and densities in comparison with untreated houses at 18 months post-intervention (Figure 4): numbers of houses positive for any developing stage (OR=0.44, 95% CI: 0.26–0.75), number of houses with larvae (OR=0.44, 95% CI: 0.26–0.75), number of larvae per house (IRR=0.36, 95% CI: 0.20–0.66), houses with pupae (OR=0.44, 95% CI: 0.23–0.82), number of pupae per house (IRR=0.22, 95% CI: 0.08–0.57) and numbers of pupae per person (IRR=0.33, 95% CI: 0.13–0.82). At 24 months, post-intervention significant reductions were found in immature density (i.e., number of larvae per house (IRR=0.33, 95% CI: 0.13–0.83), number of pupae per house (IRR=0.26, 95% CI: 0.10–0.68), and number of pupae per person (IRR=0.30, 95% CI: 0.10–0.88).

Right after the implementation of the TT intervention, all the *Stegomyia* indices and PPI showed a significant difference between intervention and control clusters (Figure 5). However, significance was transient over time, with water-holding container immatures per house and immatures per container being the only statistically significant value in the following survey (Figure 5).

### Temporal persistence of interventions

**Figure 6.**
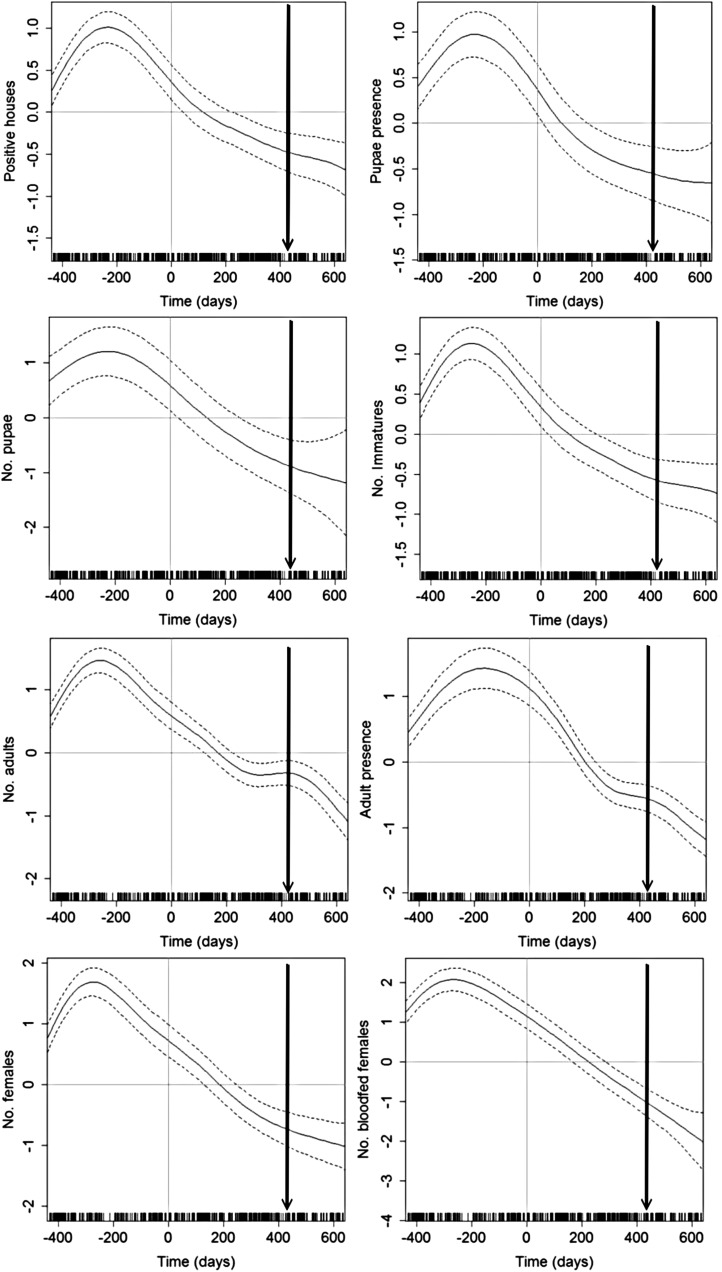
Predicted values for the best generalized additive mixed model (GAMM) showing the association between the time since long-lasting insecticidal net screens (LLIS) installation ($f\;(t_{i}))$ and each entomologic indicator. Horizontal line shows the area of no difference and vertical line the time when LLIS were installed.

Table 2 shows the parameter value and significance of non-linear parameter (f(ti)) on GAMM models estimating the association between entomologic indices and the time since LLIS installation. For all variables, a non-linear model explained better the data than a model with a linear term (ΔAIC>2). Figure 6 shows the plot of *f(t_i_)* for each entomologic indicator (immatures and adults). The y-axis can be interpreted as the effect of time since LLIS installation on each entomologic measure. When the predicted value and its 95% credible interval are negative, it means that there is a protective effect of LLIS for that factor. In all cases, LLIS achieved a protective effect for at least 600 days post installation. Adult indices (presence and abundance) showed a second reduction at 500 days post intervention, coincidentally with the introduction of the TT strategy (Figure 6).

**Table 2.** Parameter value and significance of non-linear parameter (f(t_i_)) on GAMM models estimating the association between entomologic indices and the time since LLIS installation. ΔAIC represents the difference between AIC values of a model excluding (AIC_GAM_) and including (AIC_GAMM_) a random effect associated with each cluster

Life stage	Indicator	Estimated df	*F*	p	ΔAIC (AIC_GAM_ - AIC_GAMM_)
Immature	No. immatures	5.32	26.1	<0.0001	105
	No. pupae	3.33	9.5	<0.0001	60
	Positive houses	5.35	23.0	<0.0001	956
	Pupae presence	4.55	15.8	<0.0001	1026
Adult	No. females	5.13	39.5	<0.0001	25
	No. bloodfed females	6.49	43.7	<0.0001	4
	No. adults	6.49	43.7	<0.0001	23
	Presence adults	5.62	40.1	<0.0001	21
	Presence females	5.29	39.2	<0.0001	2

AIC: Akaike Information Criterion; GAM: generalized additive model; GAMM: generalized additive mixed model; LLIS: long-lasting insecticidal net screens

## Discussion

The study showed a significant and persistent impact on *Ae. aegypti* adult and immature vector populations for up to 2 years after deployment. LLIS fixed on doors and windows should provide a mechanical as well as a chemical barrier for mosquitoes. Insecticide-treated materials have been field-evaluated in numerous different settings worldwide with some degree of success against dengue vectors, when used as a physical barrier to oviposition^3,16,17^ or to reduce human-contact and provide personal protection in the home as bednets^18^ or as window/door curtains.^3,5,6,19,20^

‘Mosquito-proofing’ houses has been employed historically in places where mosquito nuisance and disease transmission are a problem.^21^ However, few studies have evaluated simple house screening/netting for dengue vectors. Manrique-Saide et al.^11^ reported in Merida, Mexico, that the presence of untreated window screening significantly decreased both the odds of having *Aedes* adult mosquitoes inside the house and of the number of females found indoors. The pyrethroid insecticide reduces the number of vectors entering the house and potentially reduces the survival of those attempting to exit.^22,23^

The combination of LLIS with TT in the most productive container types in Acapulco was successful in further reducing the number of *Aedes* pupae and consequently adult dengue vectors. Control of breeding sites, even if applied in a TT strategy, is heavily affected by the coverage, residuality and water availability by rainfall or human practices.^24,25^ Nevertheless, the effect in Acapulco was achieved because TT was applied in the largest coverage possible and at least every two months.

The effect of controlling containers that are productive all the year round such as water tanks and metal drums, has alone a long-term effect in vector density, both as immatures and adults.^9^ Indeed in Acapulco, after treating the most productive containers, we observed a cumulative effect of the combined intervention particularly pronounced during the rainy season.

The house is an important place for human–vector contact. The prevention of human–vector contact is necessary to interrupt the dengue transmission cycle.^26^ Protection against mosquito bites and disease transmission with mosquito netting in houses has been historically observed as a fundamental technique of malaria control in the early 1900s.^21^ Protecting houses with screens has been shown to be effective in reducing malaria transmission^27–29^ and also to be a well-appreciated and sustainable vector control measure.^30^ Our study has shown that this is also true for dengue.

Control of dengue vector density at the household level and cluster level in Acapulco was notable, but it has still to be shown that this measure reduces dengue transmission and incidence. Mexican authorities have shown their interest in this approach to dengue control and offered their support by implementing a large scale study to show the impact of the measure on dengue incidence. This is now in preparation.

### Conclusions

The combination of long-lasting insecticidal screens fitted to external windows and doors and targeted treatment of the most productive *Ae. aegypti* breeding sites can impact significantly on dengue vector populations and sustain that impact for up to 24 months.
